# Can men with atrial fibrillation really rest easy with a CHA_2_DS_2_-VASc score of 0?

**DOI:** 10.1186/s12872-019-1150-z

**Published:** 2019-07-26

**Authors:** Chen-Di Cheng, Xiang Gu, Hong-Xiao Li, Ruo-Yu Duan, Lei Sun, Yi Zhang, Zheng-Yu Bao, Jian-Hua Shen, Fu-Kun Chen, Ye Zhu

**Affiliations:** 1 Xiang-Ya Medical College of Central South University, Changsha, 410008 China; 2 Clinical Medical College, Yangzhou University, Yangzhou, 225001 Jiangsu China; 3 Department of Cardiology, Northern Jiangsu Province People’s Hospital, Yangzhou, 225001 Jiangsu China; 4 Clinical Medical College, Dalian Medical University, Dalian, 116044 Liaoning China

**Keywords:** Atrial fibrillation, Ischemic stroke, Endurance exercise, CHA_2_DS_2_-VASc score, Oral anticoagulation

## Abstract

**Background:**

Atrial fibrillation (AF) significantly increases the risk of ischemic stroke depending on various risk factors. The CHA_2_DS_2_-VASc score is used widely to improve stratification of AF-related stroke to identify for whom anticoagulation could be safely withheld. As upstream therapy, the management of lifestyle for AF and related stroke prevention has been ongoing for past decades.

**Case presentation:**

A 56-year-old male was taken to our hospital because of acute ischemic stroke. Without intracranial vascular malformation and angiostenosis, two small emboli were successfully taken out from the left middle cerebral artery by mechanical thrombectomy. During the hospitalisation, no apparent abnormalities were found in various laboratory tests, echocardiogram or the coronary computed tomography angiography. However, asymptomatic paroxysmal AF was first diagnosed and was presumed to be responsible for his stroke. Noticeable, he was always in good fitness benefiting from the formed good habits of no smoking and drinking. With a CHA_2_DS_2_-VASc score of 0, he had no history of any known diseases or risk factors associated with AF and related stroke. Instead of lacking exercise, he persisted in playing table tennis faithfully 3–4 times a week and 2–3 h each time over the past 30 years, and, in fact, has won several amateur table tennis championships.

**Conclusion:**

In view of the possible pathophysiological mechanisms resulting from the long-term vigorous endurance exercise, it may be a potential risk factor for developing AF and even for subsequent stroke. Not merely should strengthen the screening for AF in specific individuals as sports enthusiasts, but the necessity of oral anticoagulant for those with a CHA_2_DS_2_-VASc score of 0 might deserve the further investigation.

**Electronic supplementary material:**

The online version of this article (10.1186/s12872-019-1150-z) contains supplementary material, which is available to authorized users.

## Background

Atrial fibrillation (AF), as the most common tachyarrhythmia, confers a 5-fold higher risk of ischemic stroke (IS) and not only increased the disability rate and mortality but also resulted in increased healthcare use and cost [1, 2]. Benefiting from a significant reduction in thromboembolic risk, oral anticoagulant (OAC) therapy is recommended for all AF patients except those considered at “true low risk” of IS, that is, with a CHA_2_DS_2_-VASc (congestive heart failure, hypertension, age 75 years or older, diabetes mellitus, previous stroke or transient ischemic attack, vascular disease, age 65 to 74 years, female) score of 0 [3]. Nevertheless, more strategies are needed without any hesitation to lower the risk of AF development and improve related stroke risk stratification. Accordingly, there has been increasing focus on the role of individual lifestyle in the management of patients with AF.

## Case presentation

**Fig. 1:**
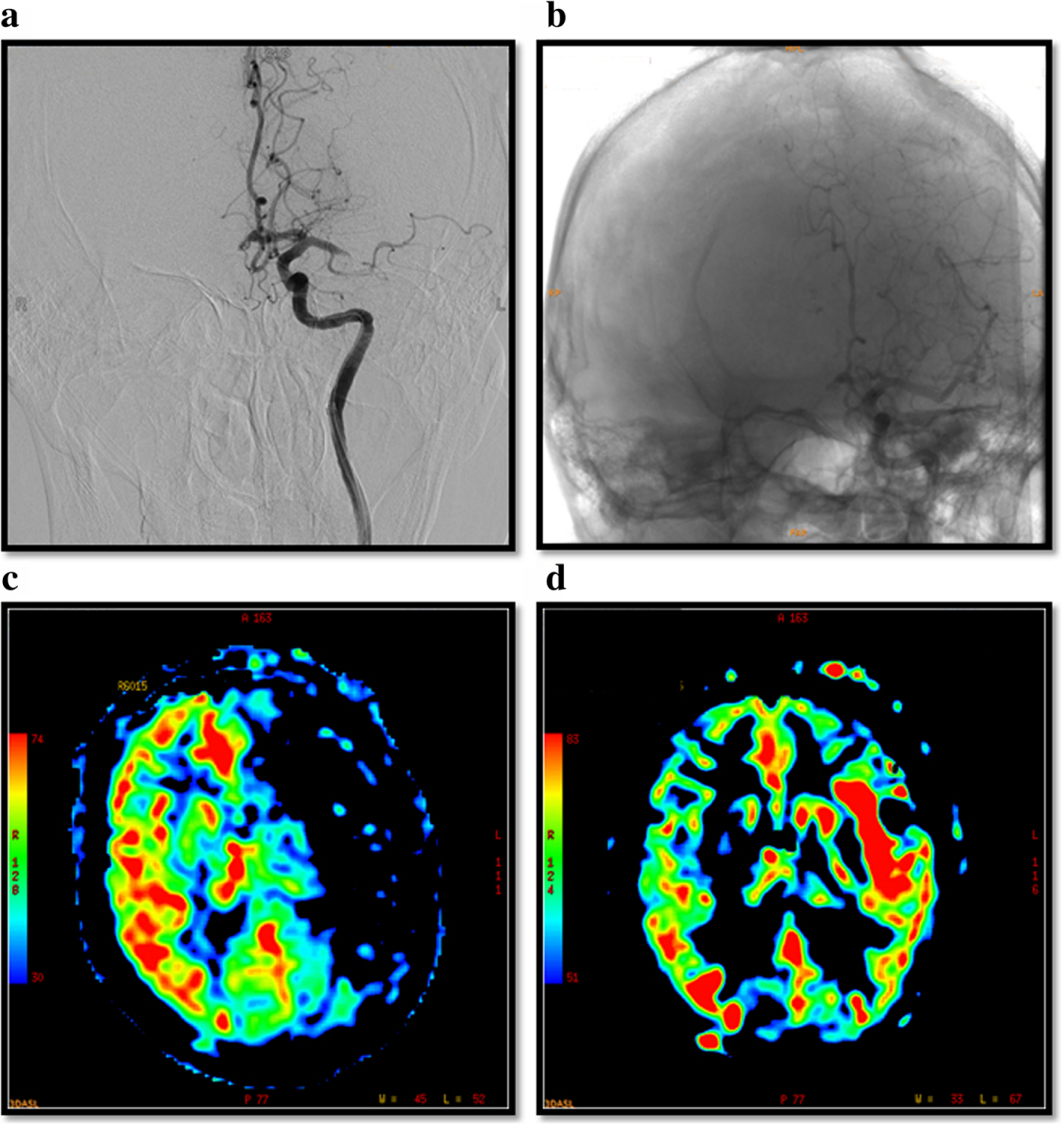
Changes of cerebral blood flow pre (**a, c**) and post (**b**, **d**) mechanical thrombectomy

A 56-year-old male was taken to our hospital due to a sudden syncope and right hemiplegia within 2 hrs. Without abnormalities detected by brain computed tomography (CT) scan, magnetic resonance perfusion imaging of arterial spin labeling showed that the perfusion of left hemisphere was significantly decreased (Fig. 1c), which was diagnosed with acute IS. CT angiography of the head and neck then suggested occlusion of the left middle cerebral artery M2 segment. After intravenous administration of recombinant tissue plasminogen activator, the result of digital subtraction angiography (DSA) (Fig. 1a) was consistent with head CT angiography. Then, the mechanical thrombectomy was performed immediately, and two small emboli were successfully taken out from the left middle cerebral artery. As expected, the repeated DSA showed the obvious recovery of cerebral blood flow without intracranial vascular malformation and angiostenosis (Fig. 1b, d).

During the hospitalisation, no apparent abnormalities found in Various laboratory tests, including blood cell analysis, blood lipid, blood sugar, renal and thyroid function, et al. Structural and congenital heart disease were excluded, and nothing abnormal could be found by echocardiogram [see Additional file 1] and cardiac enhanced CT. The asymptomatic paroxysmal AF was first recorded with the longest AF duration lasted more than 1 h and the shortest only 20 s. The long-term electrocardiograph monitoring showed that the average heart rate (HR) of 48 h was 56 beats per minute and the slowest only 36 beats per minute [see Additional file 2]. Mentionable, benefiting from the formed good habits of no smoking and drinking, he was always in good health and had none of any known diseases which were associated with AF and related stroke. Instead of lacking exercise, he persisted in playing table tennis faithfully 3–4 times a week and 2–3 h each time over the past 30 years and won several amateur table tennis championships. One month later, he was discharged smoothly through an aggressive rehabilitation program. Besides OAC therapy, it was advised that both the intensity and program of exercise should be varied and modified. Until now, he has been in a better health condition without recurrent AF and stroke since catheter ablation was performed half a year ago.

## Discussion and conclusions

Several possible known causes of the stroke, such as structural heart disease, vascular malformation and angiostenosis, abnormal medication history, were ruled out, and the potentially fatal IS of this patient was presumed to be his AF. Though the patient with non-valvular AF had none of the comorbidities or risk factors assessed by the CHA_2_DS_2_-VASc score, which means he was at “true low risk” of related IS and it was unnecessary for him to receive OAC therapy [3]. But he still suffered from such potentially fatal IS. Then questions remain: Is the long-lasting endurance activity the culprit? Is it unnecessary for asymptomatic adults to strengthen AF screening? For AF males with a CHA_2_DS_2_-VASc score of 0, could they really rest easy without anticoagulant therapy?

**Fig. 2:**
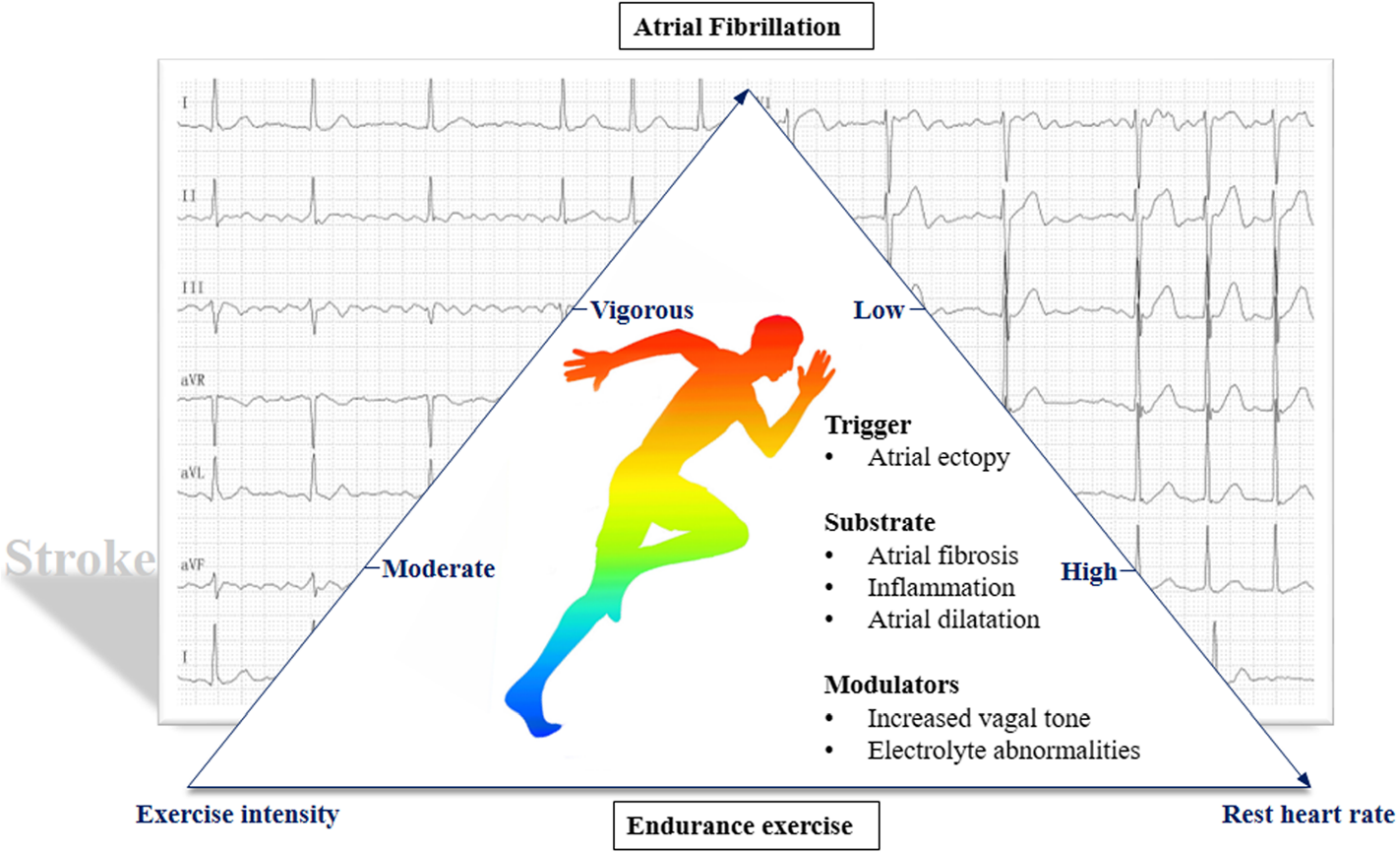
The ignored risk of AF and related stroke hidden in endurance exercise. With exercise intensity increased unduly, the vulnerability to AF and related stroke may increase relying on the possible mechanisms. AF: atrial fibrillation

Several established risk factors, including age, structural heart disease, hypertension and diabetes mellitus, are known to be strongly associated with the AF development. Other non-cardiac conditions, such as alcohol consumption and smoking, have also been described [4]. By contrast, the benefits of moderate physical activity in controlling cardiovascular risk factors are irrefutable. However, mounting evidence suggests that long-lasting endurance training may be a potential contributor to AF development since a U-shaped relationship between physical activity and incident AF was presented [5, 6]. The strenuous endurance exercise-associated AF was defined as ‘PAFIYAMA’ (‘paroxysmal AF in young and middle-aged athletes’) by Sanchis Gomar et al. [7]; because it usually starts as paroxysmal AF, frequently affecting young and middle-aged athletes, especially males.Several possible pathophysiological mechanisms have been suggested to explain the increased vulnerability to AF in athletes depending on triggers, substrates and modulators [8, 9], though the exact mechanism remains elusive. Rest HR, serving as an alternative measure of exercise intensity, might be an effective predictor of incident AF induced by physical activity due to the risk of AF increased with decreasing HR [10] (Fig. 2).

Recently, the US Preventive Services Task Force suggests it is unnecessary to screen for AF with an electrocardiography (ECG) in asymptomatic adults. Because the current evidence may be insufficient to assess the net benefits for the general population [11]. Actually, approximately 20% of patients who suffered from AF-related stroke are first diagnosed with AF at the time of stroke [12]. It will be more attractive to identify AF before related IS in view of the disastrous consequences. More attention should be paid to primary prevention which has largely untapped potential for improving AF management. Therefore, for the special population who have a long-lasting experience of endurance exercise, such as athletes, sports enthusiasts, manual workers and soldiers, particularly those with lower HR, it is worth to strengthen the screening for AF. Not merely the long-lasting ECG monitoring may be helpful but also the regular echocardiography is useful to evaluate the left atrial structure.

More importantly, for patients who were undergoing the chronic vigorous aerobic activity, if paroxysmal AF was identified fortunately before a IS occurs, could they really rest easy with a CHA_2_DS_2_-VASc score of 0? In the general population, the CHA_2_DS_2_-VASc score improved IS risk stratification dramatically and was widely used to identify those who can safely forego OAC. While the net clinical benefit of OAC is clearly established for patients with AF at high risk for stroke (CHA_2_DS_2_-VASc score ≥ 2), the decision to anticoagulate for patients with a CHA_2_DS_2_-VASc score of one still represents a matter of controversy in international guidelines [13]. Lip et al. and Fauchier et al. [14, 15] seemed to prefer the approach advocated in the stroke prevention guidelines from the ESC 2016, and reported that OAC was recommended for all AF patients except for those with a CHA_2_DS_2_-VASc score of 0 (beyond gender) [3]. In addition, antithrombotic therapy was taken into consideration for lone AF patients who usually have a low CHA_2_DS_2_-VASc score, but is only a Class II b recommendation (Level C) in JCS 2013 [16]. By contrast, benefiting from the long-term physical activity, the athletes or sports enthusiasts are usually healthier, and without significant comorbidities, most of them had a lower score compared to the general population. There is no evidence that stroke risk in athletes and non-athletes with the same CHA2DS2-VASc score are the same. In other words, do athletes or sports enthusiasts with low CHA_2_DS_2_-VASc score confer the same “true low risk”as general population? A large cohort study by Myrstad et al. [17] demonstrated that a high prevalence of stroke remained among veteran skiers with AF; furthermore, skiers with AF were slightly younger and had less co-morbidity than their counterparts from the general population. Another observational prospective study by Marco Proiett et al. [18] suggested that no significant difference in rates of stroke between different self-reported physical activity level. However, the level of physical activity was self-reported according to the time by patients, irrespective of the type of activity. Among 4.7% of patients who had reported intense physical activity, 83.5% of them had CHA_2_DS_2_-VASc class score ≥ 1, and most of them had already received antithrombotic therapy. It may help explain why the same stroke rate was found in different physical activity level. Thus, the risk of AF-related stroke need more evidences to describe in athletes with CHA_2_DS_2_-VASc of 0. Noticeably, several studies have reported the potential relationship between left atrial structures and the risk of IS in AF, especially the left atrial fibrosis [19], which was also considered as a crucial potential mechanistic contributor of AF induced by long-lasting endurance exercise [20]. Thus, we suppose that for the long-term vigorous endurance exercisers, the risk of AF-related stroke should be evaluated by more strategies and tools beyond the CHA2DS2-VASc score. Factors such as impaired renal function, obstructive sleep apnea, and biochemical parameters can be used to predict adverse thromboembolic events [21, 22]. Transesophageal echocardiography is more accurate in assessing left atrial appendage morphology and function and for the detection of intracardiac thrombi [23]. However, the net clinical benefit balancing stroke reduction against major bleeding remains to further investigation because of the high risk of traumatic bleeding. The catheter ablation for AF may be worth to take into consideration.

In conclusion, in view of the possible U-shaped relationship between physical activity and incident AF, the long-term vigorous endurance exercise may be a potential risk factor for developing AF and even for AF-related stroke based on several possible pathophysiological mechanisms. For such special population, especially those with lower HR, it is worth to strengthen the AF screening. Once identified, the exercise intensity should be first adjusted scientifically, then the risk of AF-related stroke should be evaluated by more strategies and tools. The necessity of OAC therapy for those with the CHA2DS2-VASc score of 0 might deserve further investigation.

## Additional files

Additional file 1: Images about echocardiogram. Structural heart disease was ruled out and nothing abnormal could be found in cardiac structural and function by echocardiogram. (PNG 1100 kb)

Additional file 2: Images about routine electrocardiography. The paroxysmal AF was recorded with the longest AF duration lasted more than 1 h and the shortest only 20 s. The long-term electrocardiogram monitoring showed that the average heart rate was 56 beats per minutes and the slowest only 36 beats per minutes. (PNG 1830 kb)

## Data Availability

All the data supporting our findings is contained within the manuscript.

## Abbreviations

AF: Atrial fibrillation
CHA_2_DS_2_-VASc: Congestive heart failure, hypertension, age 75 years or older, diabetes mellitus, previous stroke or transient ischemic attack, vascular disease, age 65 to 74 years, female
CT: Computed tomography
DSA: Digital subtraction angiography
ECG: Electrocardiography
HR: Heart rate
IS: Ischemic stroke
OAC: Oral anticoagulant

## References

[1] Lip GY, Tse HF, Lane DA (2012). Atrial fibrillation. Lancet..

[2] Vigdis V, Carla T, Steven E (2016). Association between atrial fibrillation, anticoagulation, risk of cerebrovascular events and multimorbidity in general practice: a registry-based study. BMC Cardiovasc Disord.

[3] Kirchhof P, Dipak K, Casadei B (2016). 2016 ESC guidelines for the management of atrial fibrillation developed in collaboration with EACTS: the Task Force for the management of atrial fibrillation of the European Society of Cardiology (ESC). Eur Heart J.

[4] Schnabel RB, Yin X, Gona P (2015). 50 year trends in atrial fibrillation prevalence, incidence, risk factors, and mortality in the Framingham heart study: a cohort study. Lancet.

[5] Mohanty S, Mohanty P, Tamaki M (2016). Differential Association of Exercise Intensity with Risk of atrial fibrillation in men and women: evidence from a meta-analysis. J Cardiovasc Electrophysiol.

[6] Baldesberger S, Bauersfeld U, Candinas R (2007). Sinus node disease and arrhythmias in the long-term follow-up of former professional cyclists. Eur Heart J.

[7] Sanchis-Gomar F, Perez-Quilis C, Lippi G (2017). Atrial fibrillation in highly trained endurance athletes — description of a syndrome. Int J Cardiol.

[8] D'Ascenzi F, Cameli M, Ciccone MM (2015). The controversial relationship between exercise and atrial fibrillation: clinical studies and pathophysiological mechanisms. J Cardiovasc Med.

[9] Mont L, Elosua R, Brugada J (2008). Endurance sport practice as a risk factor for atrial fibrillation and atrial flutter. Europace.

[10] Morseth B, Graffiversen S, Jacobsen BK (2016). Physical activity, resting heart rate, and atrial fibrillation: the Tromso study. Eur Heart J.

[11] Preventive Services Task Force US (2018). Screening for atrial fibrillation with electrocardiography US Preventive Services Task Force recommendation statement. JAMA.

[12] Healey JS, Connolly SJ, Gold MR (2012). Subclinical atrial fibrillation and the risk of stroke. N Engl J Med.

[13] January CT, Wann LS, Alpert JS (2014). ACC/AHA Task Force members. 2014 AHA/ACC/HRS guideline for the management of patients with atrial fibrillation: executive summary: a report of the American College of Cardiology/American Heart Association Task Force on practice guidelines and the Heart Rhythm Society. Circulation.

[14] Lip GY, Skjøth F, Rasmussen LH (2015). Oral anticoagulation, aspirin, or no therapy in patients with nonvalvular AF with 0 or 1 stroke risk factor based on the CHA2DS2-VASc score. J Am Coll Cardiol.

[15] Fauchier L, Clementy N, Bisson A (2016). Should atrial fibrillation patients with only 1 nongender-related CHA2DS2-VASc risk factor be anticoagulated?. Stroke.

[16] JCS Joint Working Group (2014). Guidelines for pharmacotherapy of atrial fibrillation (JCS 2013). Circ J.

[17] Myrstad M, Aarønæs M, Graff-Iversen S (2016). Physical activity, symptoms, medication and subjective health among veteran endurance athletes with atrial fibrillation. Clin Res Cardiol.

[18] Proietti M, Boriani G, Laroche C (2017). Self-reported physical activity and major adverse events in patients with atrial fibrillation: a report from the EURObservational research Programme pilot survey on atrial fibrillation (EORP-AF) general registry. Europace.

[19] Daccarett M, Badger TJ, Akoum N (2011). Association of left atrial fibrosis detected by delayed-enhancement magnetic resonance imaging and the risk of stroke in patients with atrial fibrillation. J Am Coll Cardiol.

[20] Guasch E, Benito B, Qi X (2013). Atrial fibrillation promotion by endurance exercise: demonstration and mechanistic exploration in an animal model. J Am Coll Cardiol.

[21] Savino JA, Halperin JL (2016). Should patients with atrial fibrillation and 1 stroke risk factor (CHA2DS2-VASc score 1 in men, 2 in women) be anticoagulated? The CHA2DS2-VASc 1 conundrum: decision making at the lower end of the risk spectrum. Circulation.

[22] Szymanski FM, Lip GY, Filipiak KJ (2015). Stroke risk factors beyond the CHA2DS2-VASc score: can we improve our identification of “high stroke risk” patients with atrial fibrillation?. Am J Cariol.

[23] Di Biase L, Santangeli P, Anselmino M (2012). Does the left atrial appendage morphology correlate with the risk of stroke in patients with atrial fibrillation? Results from a multicenter study. J Am Coll Cardiol.

