# Supplementary figures and images for: Can men with atrial fibrillation really rest easy with a CHA2DS2-VASc score of 0?
Source: BMC Cardiovasc Disord. 2019 Jul 26;19:178. doi: 10.1186/s12872-019-1150-z (PMC6660687; doi:10.1186/s12872-019-1150-z)

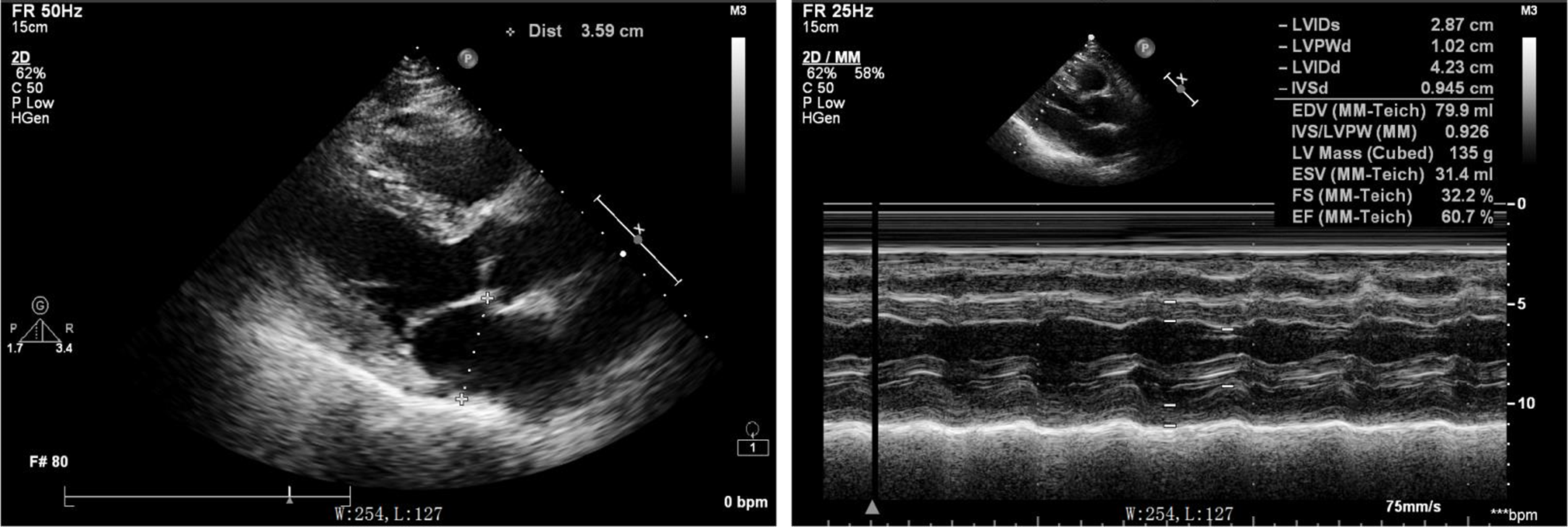

Supplement: Supplementary file 1 — Images about echocardiogram. Structural heart disease was ruled out and nothing abnormal could be found in cardiac structural and function by echocardiogram. (PNG 1100 kb) [file 12872_2019_1150_MOESM1_ESM.png]

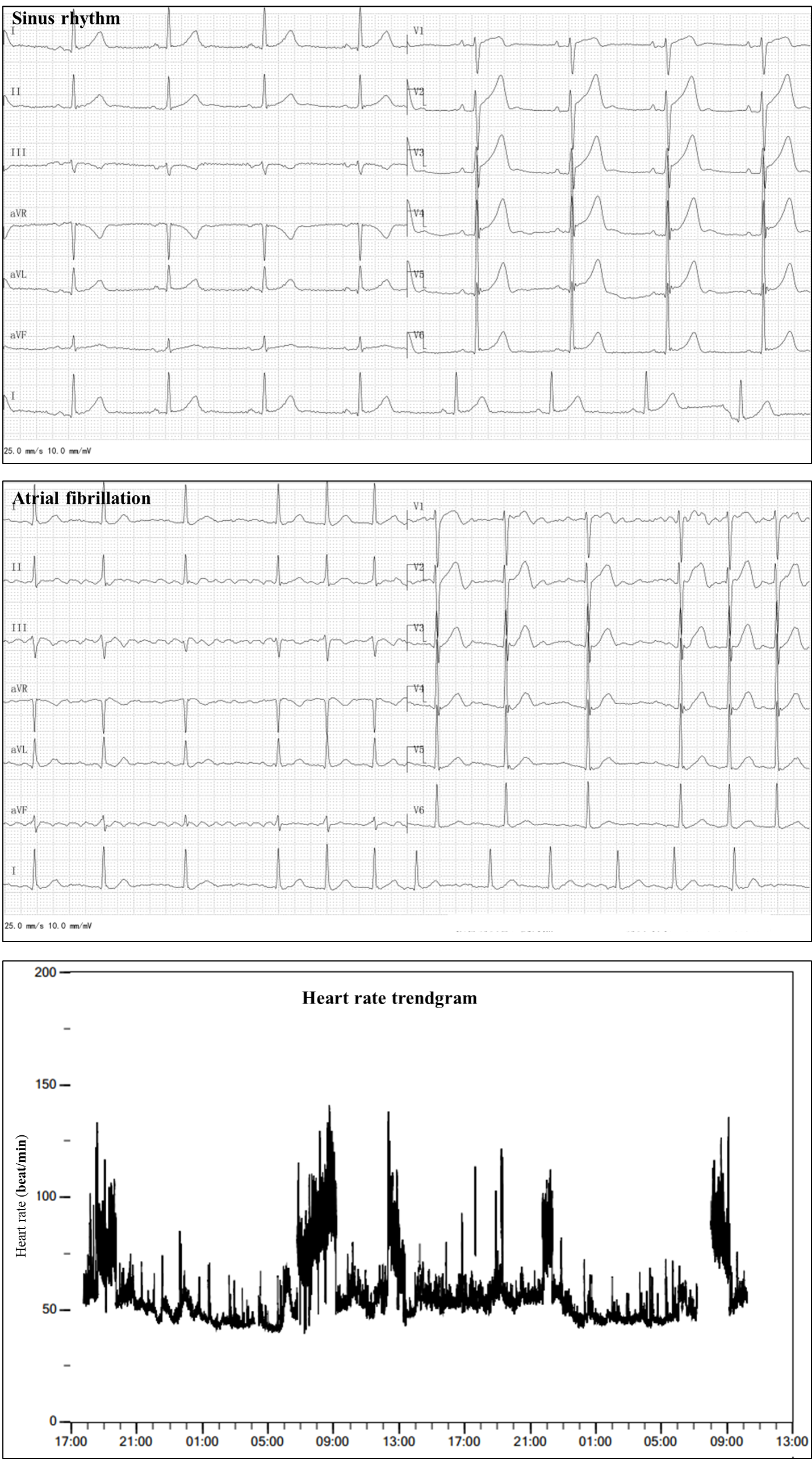

Supplement: Supplementary file 2 — Images about routine electrocardiography. The paroxysmal AF was recorded with the longest AF duration lasted more than 1 h and the shortest only 20 s. The long-term electrocardiogram monitoring showed that the average heart rate was 56 beats per minutes and the slowest only 36 beats per minutes. (PNG 1830 kb) [file 12872_2019_1150_MOESM2_ESM.png]
